# A subserosal, pedunculated, multilocular uterine leiomyoma with ovarian tumor-like morphology and histological architecture of adenomatoid tumors: a case report and review of the literature

**DOI:** 10.1186/s13256-016-1167-1

**Published:** 2016-12-20

**Authors:** Kenji Yorita, Yu Tanaka, Koki Hirano, Yuka Kai, Kaoru Arii, Kimiko Nakatani, Satoshi Ito, Toshiya Imai, Masaharu Fukunaga, Naoto Kuroda

**Affiliations:** 1 Department of Diagnostic Pathology, Japanese Red Cross Kochi Hospital, 2-13-51, Shinhonmachi, Kochi-city, Kochi 780-8562 Japan; 2 Department of Obstetrics and Gynecology, Japanese Red Cross Kochi Hospital, 2-13-51, Shinhonmachi, Kochi-city, Kochi 780-8562 Japan; 3 Department of Internal Medicine, Japanese Red Cross Kochi Hospital, 2-13-51, Shinhonmachi, Kochi-city, Kochi 780-8562 Japan; 4 Department of Radiology, Japanese Red Cross Kochi Hospital, 2-13-51, Shinhonmachi, Kochi-city, Kochi 780-8562 Japan; 5 Minaminokaze Clinic, 3-7-1, Sakaemachi, Kochi-city, Kochi 780-0061 Japan; 6 Department of Pathology, Shinyurigaoka, General Hospital, 255, Furusawa, Asao-ku, Kawasaki-city, Kanagawa 215-0026 Japan

**Keywords:** Leiomyoma, Cystic degeneration, Hydropic degeneration, Adenomatoid tumor

## Abstract

**Background:**

Uterine leiomyomas are common uterine tumors, and typical cases of leiomyoma are easily diagnosed by imaging study. However, uterine leiomyomas are often altered by degenerative changes, which can cause difficulty and confusion in their clinical diagnosis. We describe the 17th reported case of a uterine leiomyoma clinically diagnosed as an ovarian tumor; however, the present case shows the most detailed radiological evaluation, including contrast-enhanced magnetic resonance imaging. We first show that a uterine leiomyoma can histologically mimic an adenomatoid tumor.

**Case presentation:**

A 47-year-old premenopausal, nulliparous Japanese woman with a history of type 2 diabetes mellitus, hypertension, and hyperlipidemia had lower abdominal pain. Ultrasonography confirmed a 6-cm mass in the right-sided space of the pelvic cavity. Magnetic resonance imaging evaluation showed that a multilocular mass was present near the uterus, and a mucinous ovarian tumor was considered. Emergency surgery due to acute abdomen was performed under the diagnosis of pedicle torsion of the ovarian tumor. During surgery, a pedunculated uterine mass without stalk torsion was seen. The mass grossly contained serous and hemorrhagic fluids in the cavities, and pathology examination confirmed that the mass was a leiomyoma with hydropic and cystic degeneration. Anastomosing thin cord-like arrangements of the leiomyoma cells mimicked the architecture of adenomatoid tumors. The tumor cells were positive for the microphthalmia transcription factor but negative for other melanoma markers. Three days postoperatively, she was discharged without sequelae.

**Conclusions:**

Marked intratumoral deposition of fluids may induce the multilocular morphology of a tumor, and the cellular arrangement of the tumor cells with hydropic degeneration mimicked an adenomatoid tumor in this case. Clinicians need to be aware that a subserosal leiomyoma with cystic and hydropic degeneration can mimic an ovarian tumor, and pathologists should be aware that such leiomyomas can mimic adenomatoid tumors. Additionally, perivascular epithelioid cell tumors should not be diagnosed only based on its immunoreactivity for the microphthalmia transcription factor.

## Background

Typical cases of leiomyoma are easy to diagnose radiologically. However, uterine leiomyomas are commonly altered by degenerative changes, which can cause the misdiagnosis of tumors. In the present case, a submucosal, pedunculated leiomyoma mimicked an ovarian tumor with degenerative changes. Until now, 16 cases of a leiomyoma with the clinical diagnosis of an ovarian tumor (an ovarian tumor-like leiomyoma) have been reported [1–15]. We review the 16 cases and show the clinicopathological characteristics of ovarian tumor-like leiomyomas. We also describe the unique features of the present case.

## Case presentation

**Fig. 1:**
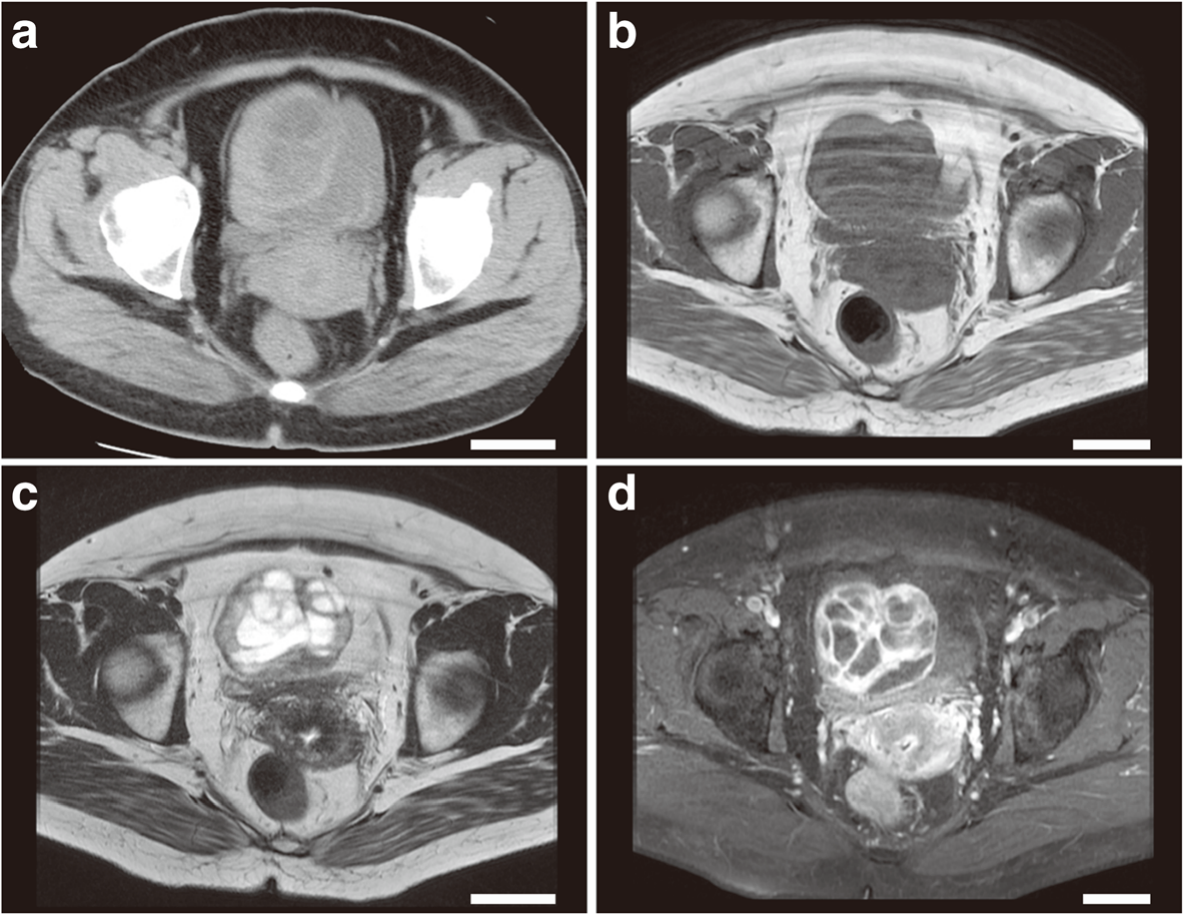
Radiographic images of the tumor. (**a**) An axial non-enhanced computed tomography image. A nodular lesion was seen adjacent to the uterus. The mass had low-density areas, and the peripheral and septal portions were isodense relative to the uterus. (**b**–**d**) Magnetic resonance images in the axial plane. The tumor had an intermediate to low signal intensity on a T1-weighted image; (**b**) a high signal intensity, particularly of the cavities, on a T2-weighted image (**c**); and a multilocular enhancement pattern on a contrast-enhanced, fat-suppressed T1-weighted image (**d**). The *white bars* represent 5 cm

A 47-year-old premenopausal, nulliparous Japanese woman was referred to our hospital because of an abdominal mass and a symptom of abdominal pain. She had a history of type 2 diabetes mellitus, hypertension, hyperlipidemia, and fatty liver, and she was treated for the former two conditions. No history of surgery was found. Her vital signs were unremarkable, and rebound tenderness was absent. Transvaginal ultrasonography (US) confirmed a 77 × 60 × 51-mm multilocular mass near the right side of the uterus, and ascites was also seen. Since the right ovary could not be seen, a right ovarian tumor was considered. Various tumor markers, including the carcinoembryonic antigen, cancer antigen 72–4, carbohydrate antigen 19–9, and cancer antigen 125 (CA-125) were within normal limits. Bloody ascites was obtained, but abnormal cells were not confirmed cytologically. A computed tomography (CT) scan showed that the mass was present near the uterus, and it appeared to have a heterogeneous appearance with isodensity to hypodense areas relative to the uterine myometrium (Fig. 1a). A magnetic resonance imaging (MRI) scan showed the heterogeneous mass (Fig. 1b-d), and it had a low signal intensity on T1-weighted images (T1WIs) (Fig. 1b). T2WIs (Fig. 1c) showed that the mass had high signal intensity areas, and the other portions were slightly hyperintense relative to the uterine myometrium. A fat-saturated, gadolinium-enhanced T1WI (Fig. 1d) showed that the peripheral and septum-like portions of the mass were well enhanced, and no enhancement was seen in the hyperintense portions on T2WIs. No fat component was observed. Thus, the mass was considered a multilocular cystic lesion containing fluid materials in the cavities. On CT and MRI scans, the right ovary was not visible, but a left swollen ovary was found. Thus, a right ovarian mucinous tumor was considered. Aside from the mass, a 15-mm intramural, solid, demarcated nodule was seen in the uterine body. Her abdominal pain gradually decreased; additionally, 2 weeks after the onset of abdominal pain, the mass decreased in size to 50 × 47 × 46 mm, and no ascites was seen by transvaginal US. Two months after the onset of abdominal pain, the mass slightly enlarged to 60 × 50 × 50 mm without apparent symptoms, and surgical resection was planned. One week later, sudden abdominal pain resulted in emergency surgery under the diagnosis of torsion of the pedicle of the ovarian tumor. During surgery, we found a 6-cm pedunculated, round mass connected to the uterine fundus by a 1-cm narrow stalk. The mass was red and hemorrhagic, and bloody ascites was seen; however, neither torsion of the stalk nor tumor rupture was observed. A normal right ovary was seen, and the left ovary was swollen with cystic change. Thus, a subserosal leiomyoma was considered. There were no gross features of the cotyledonoid dissecting leiomyoma, which are multiple bulbous protrusions from the uterine serosa. Extirpation of the pedunculated uterine mass and cystectomy of the left ovarian lesion were performed, and our patient was discharged 3 days postoperatively without sequelae.

**Fig. 2:**
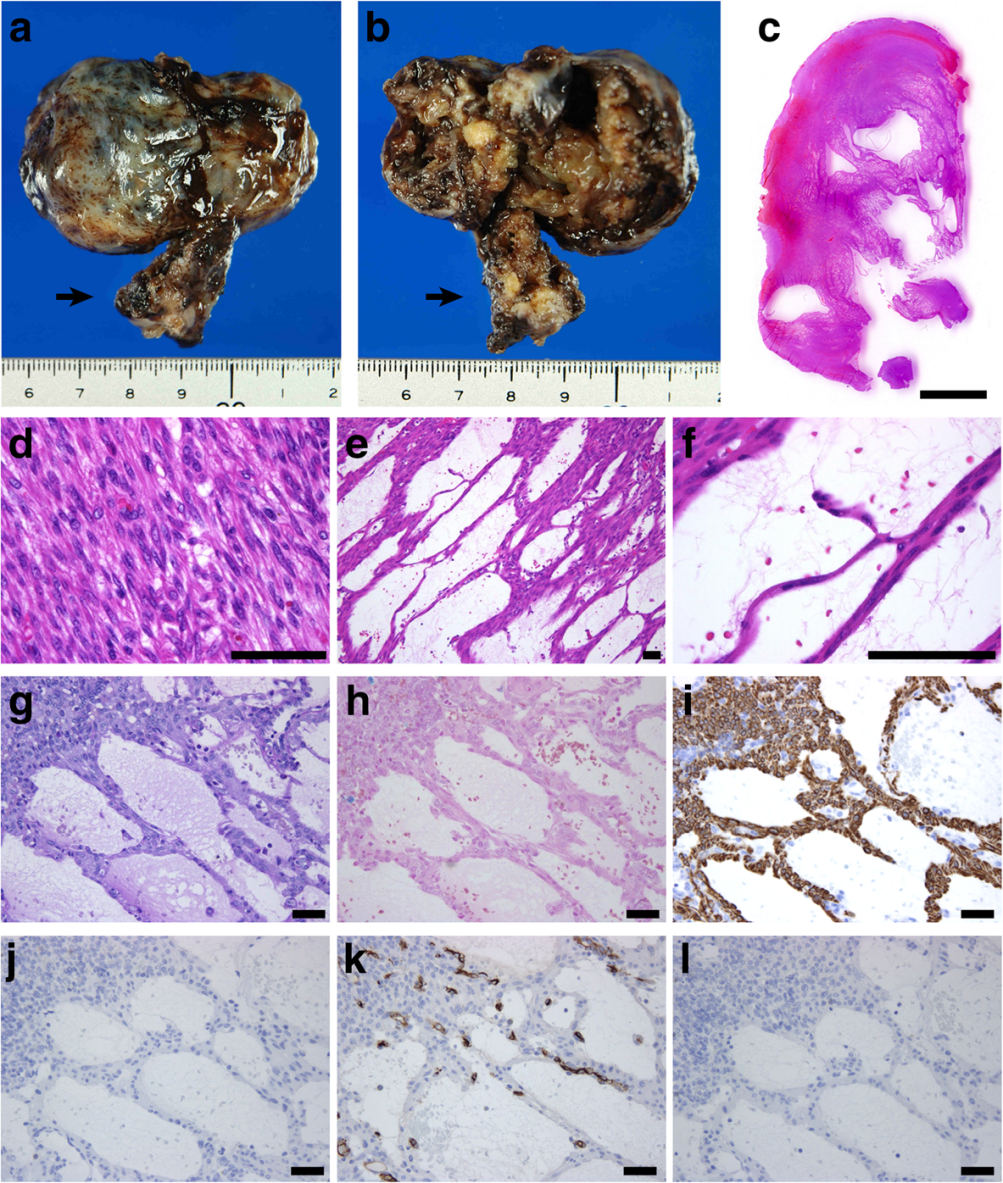
Macroscopic and microscopic findings. (**a**–**b**) The macroscopic appearance of the tumor fixed in formalin. The heterogeneously brown mass had a smooth surface (**a**), and the cut surface had a solid, cystic, hemorrhagic appearance (**b**). The tumor stalk is indicated by *an arrow*. (**c**–**f**) Microscopic findings of the hematoxylin-and-eosin-stained sections. A low-magnification figure of the cut surface of the tumor showing a solid mass with various sized irregular cystic spaces (**c**). The solid portion of the tumor indicated a typical leiomyoma because of the presence of the interlacing arrangement of plump spindle cells (**d**). Hydropic and cystic degeneration of the leiomyoma was seen (**e**–**f**), including anastomosing, thin cords of the leiomyoma cells. (**g**–**h**) Special staining of the degenerated portion of the tumor. No mucinous materials were seen in the cystic spaces in sections stained by periodic acid-Schiff with diastase treatment (**g**) and Alcian blue (**h**). (**i**–**l**) Immunohistochemical finding of the tumor. The spindle cells were positive for desmin (**i**), and no lining cells were positive for calretinin (**j**), CD34 (**k**), or D2-40 (**l**). The *black bar* in **c** represents 5 mm, and the *black bars* in **d**–**l** represent 100 μm

Macroscopically, the formalin-fixed uterine tissue was dark and red (Fig. 2a-b), and the cut surface was solid with a cystic appearance and brownish or serous fluids. The peripheral portion of the uterine mass was particularly seen as a solid component. A leiomyoma with red degeneration was grossly considered. The cystic cavities (Fig. 2c), which were variable in size and shape, tended to be centrally accumulated in the mass. Microscopically, the cystic lesion of the left ovary was a hematoma with no neoplasia. The solid and cystic portions of the uterine mass consisted of fascicular and interlacing bland spindle cells with eosinophilic cytoplasm (Fig. 2d-f). The tumor cells had a uniform shape and scarce mitoses. Coagulation necrosis was absent, although the gross morphology of the tumor suggested red degeneration, which indicates coagulation necrosis of leiomyoma cells. Particularly, the peripheral portion of the mass demonstrated a typical feature of a leiomyoma (Fig. 2d). No apparent lining cells were seen on the cavities. In microscopically cystic lesions, thin cords of the spindle cells irregularly anastomosed to each other with serous and hemorrhagic fluids in the background (Fig. 2e-f). The anastomosing, thin, cord-like arrangement of the spindle cells appeared to be thread-like bridging strands, which is a pathological clue of adenomatoid tumors. The tumor lacked an infiltrative border and perinodal hydropic change. The pathological diagnoses included a leiomyoma and adenomatoid tumor. A perivascular epithelioid cell tumor (PEComa) could not be ruled out, because intratumoral abnormal vessels may be present. A periodic acid-Schiff stain with diastase treatment (Fig. 2g) and Alcian blue stain (Fig. 1-h) confirmed that the cystic cavities had no mucin. No iron deposition was present in the tumor according to the results of Berlin blue staining, although some hemorrhagic lesions were noted. Immunohistochemically, the spindle cells were positive for α-smooth muscle actin, desmin (Fig. 2i), the estrogen receptor, and progesterone receptor, and they were negative for cytokeratin 5 and calretinin (Fig. 2j). Although the tumor cells were partly positive for the microphthalmia transcription factor (MiTF), no immunoreactivity for the human melanoma black 45 and melan-A was seen. No dilated change in the intratumoral venous or lymphatic channels was observed in immunohistochemical sections with CD34 (Fig. 2k) or D2-40 (Fig. 2l). Thus, the mass was considered a subserosal, pedunculated leiomyoma with hydropic and cystic degeneration. Our patient had an additional intramural uterine tumor, and hysterectomy was performed to evaluate the uterine mass. Pathology results showed an intramural leiomyoma without cystic or hydropic degeneration. Neither malignancy nor adenomyosis was seen. Our patient had an unremarkable postoperative course.

## Discussion

Using the PubMed database, we reviewed cases of a cystic and/or multilocular leiomyoma clinically diagnosed as an ovarian tumor (ovarian tumor-like leiomyoma). Among 16 cases of leiomyomas [1–15], 12 [1, 3–11, 14] were derived from the uterus and 4 [2, 12, 13, 15] originated from a broad ligament or fallopian tube; these cases are summarized in Table 1 along with the present case. The 17 cases of an ovarian tumor-like leiomyoma had a predilection for middle-aged women (mean age 45 years, range 21–58 years) and a mean maximal size of 21 cm (range 5–40 cm). The present case had the smallest tumor among them. Acute abdomen seen in the present case may be a rare symptom in cases of an ovarian tumor-like leiomyoma, because such a symptom has only been reported in 2 [1, 8] of 16 cases. Tumor rupture may be the cause of acute abdomen in Takai *et al*.’s case [8], whereas transient torsion of the tumor stalk may be the cause of acute abdomen in our case. Preoperative serum tumor markers were evaluated in 11 [5, 6, 8–15] of 17 cases, and in 6 [6, 8, 10, 11, 14, 15] of 11 cases, only the CA-125 level was increased among the tumor markers. Two cases had an increased serum CA-125 level, which normalized after tumor resection [6, 10]. The mechanism of an abnormal serum CA-125 level in cases of pelvic leiomyomas is unclear.

**Table 1:** Reviewed cases of a leiomyoma clinically diagnosed as an ovarian tumor

First author (published year)	Age (years)	Increased tumor markers	Imaging modalities used	Tumor size (cm)	Gross morphology of the tumor	Tumor site	Pathological diagnosis	Contents of the cavities
Togashi (1986) [1]	35	NA	US, CT	NA, 4.3kg	Cystic (multilocular), pedunculated	Uterus	Leiomyoma, subserosal	Mucinous material
Togashi (1986) [1]	21	NA	CT	10	Cystic, pedunculated	Uterus	Leiomyoma, subserosal	NA
Carabias (1995) [2]	54	NA	US, CT	10	Solid and cystic (spongy cut surface)	Fallopian tube	Leiomyoma, intramural, subserosal	Fibrinous fluids
Yarwood (1999) [3]	51	NA	US	10	Cystic	Uterus	Leiomyoma, subserosal	Clear yellow fluid
Kulshrestha (2003) [4]	40	NA	US	21	Cystic and solid, pedunculated	Uterus	Leiomyoma, subserosal	Serous fluid
Low (2004) [5]	56	Normal	US, CT	19	Solid and cystic (multilocular), pedunculated	Uterus	Leiomyoma, subserosa	Yellowish fluid
Jao (2007) [6]	55	CA-125^a^	US	32	Solid and cystic (multilocular), pedunculated	Uterus	Leiomyoma, subserosal	Old bloody fluid (8L)
Dancz (2008) [7]	51	NA	US, CT, MRI	40	Cystic (multilocular), pedunculated	Uterus	Leiomyoma, subserosal	Old blood (7L)
Takai (2013) [8]	46	CA-125	CT	13	Solid and cystic (multilocular), pedunculated	Uterus	Leiomyoma, subserosal	ND (blood-stained ascites)
Aydin (2013) [9]	58	Normal	US	33	Solid and cystic (multilocular), pedunculated	Uterus	Leiomyoma, subserosal	Old blood (7L)
Gajewska (2013) [10]	40	CA-125^a^	US	25	Solid and cystic, pedunculated	Uterus	Leiomyoma, subserosal	NA
Hacivelioglu (2014) [11]	32	CA-125	US, MRI	20	Solid and cystic, pedunculated	Uterus	Leiomyoma, subserosal	NA
Bansal (2014) [12]	45	Normal	US, MRI	25	Solid and cystic (multilocular)	Broad ligament	Leiomyoma	Hemorrhagic fluid
Naz Masood (2014) [13]	43	Normal	US, MRI	19.5	Solid and cystic (multilocular)	Broad ligament	Leiomyoma	Yellow serous fluid
Karaman (2015) [14]	45	CA-125	US and MRI	30	Solid and cystic, pedunculated	Uterus	Lipoleiomyoma, subserosal	NA
Sharma (2016) [15]	50	CA-125	ND	29	Solid and cystic	Broad ligament	Cellular leiomyoma	Hemorrhagic fluid.
Present case	47	Normal	US, CT, MRI	5	Solid and cystic (multilocular), pedunculated	Uterus	Leiomyoma, subserosal	Hemorrhagic fluid

*Abbreviations*: *NA* not available, *US* ultrasonography, *CT* computed tomography, *MRI* magnetic resonance imaging

^a^Serum CA-125 level was normalized after surgery

Degenerative or secondary changes are detectable in approximately 65% of uterine leiomyomas: hyaline degeneration (63%), myxoid changes (19%), calcification (8%), cystic changes (4%), fatty metamorphosis (3%), and red degeneration (3%) [16]. Hydropic degeneration characterized by the intratumoral accumulation of edematous fluid is also known as a degeneration of uterine leiomyomas [16]. In the present case, cystic and hydropic degeneration was seen; hydropic degeneration may have induced cystic morphology, because the irregular cystic spaces were filled with serous or hemorrhagic fluid. All 16 cases of an ovarian tumor-like leiomyoma largely demonstrated degenerative changes similar to those in the present case. In 11 [1–7, 9, 12, 13, 15] of 16 cases, the contents of the cystic cavities were described, and 10 of these 11 cases had clear, yellow, or hemorrhagic fluids. One [1] of the 11 cases had mucinous materials. Thus, cystic degeneration of ovarian tumor-like leiomyomas appeared to be mostly associated with hydropic degeneration, as in the present case, and rarely associated with myxoid degeneration. The cause of degeneration was unknown. Kamat *et al*. [17] suggested that cystic degeneration of the leiomyoma represents a late stage of hyaline degeneration that could undergo liquefaction; however, no hyaline degeneration was observed in our case. Subserosal pedunculation may affect the blood supply and be associated with the pathogenesis, because the concurrent intramural leiomyoma showed neither cystic nor hydropic degeneration.

As was seen in the present case, most ovarian tumor-like uterine leiomyomas (12 cases) [1, 3–11, 14] that we reviewed were subserosal pedunculated masses, and 6 [1, 5–9] of 12 cases had a multilocular cystic morphology. All 12 ovarian tumor-like uterine leiomyomas [1, 3–11, 14] could not be clinically diagnosed as uterine leiomyomas using imaging modalities, including US, CT, and/or MRI partly because these modalities failed to detect the stalk of the tumors. The present case also resulted in a clinical diagnosis of an ovarian tumor, despite performing a more detailed radiological study than that in the aforementioned 12 cases, for which contrast-enhanced MRI was not performed. Although US is a useful modality for initially evaluating patients with a pelvic lesion, MRI is a superior modality for making a qualitative diagnosis. The present case showed that the intratumoral areas had a low signal intensity on T1WIs and a high signal intensity on T2WIs, and such MRI findings were also seen in some cases of an ovarian tumor-like leiomyoma [11–13] and cases of cellular leiomyoma or leiomyoma with hydropic degeneration [18]. Leiomyomas with cystic and hydropic degeneration show low-T1WI and high-T2WI areas with no enhancement, whereas cellular leiomyomas and leiomyomas with hydropic degeneration demonstrate enhancement on contrast-enhanced images [18]. Thus, the MRI findings of the present case were comparable with those of leiomyomas with cystic and hydropic degeneration; however, the MRI findings seemed consistent with those of an ovarian tumor, and they markedly differed from those of usual (non-degenerated) leiomyomas that show a hypointensity or isointensity compared to the normal myometrium on T1WIs and homogeneous hypointensity on T2WIs [19]. Furthermore, increased preoperative serum CA-125 may occur in ovarian tumor-like uterine leiomyomas. Thus, making the preoperative diagnosis of an ovarian tumor-like uterine leiomyoma will be very challenging.

Various mimickers of ovarian cystic tumors are non-ovarian cystic lesions of the pelvis, including a peritoneal inclusion cyst, paraovarian cyst, appendiceal mucocele, obstructed fallopian tube, cystic adenomyosis, and uterine leiomyoma [20]. In such mimickers, a multilocular morphology can be seen in peritoneal inclusion cysts, spinal meningeal cysts, tailgut cysts, and adenomyosis. Dissimilar from the present case, peritoneal inclusion cysts usually have thin walls, and spinal meningeal cysts and tail gut cysts tend to be located in the presacral region. Adenomyosis can be excluded, because the fluid contents usually show a high signal intensity on T1WI and wall of the mass usually has a low signal intensity on T2WI [20]. Thus, non-ovarian tumors of the pelvis can be ruled out in the differential diagnosis of the present case.

Pathologically, the leiomyoma in our case showed marked hydropic and cystic degeneration, and the unusual histological features required differential pathological diagnoses. First, PEComa was considered as a differential diagnosis, because the tumor cells were partially positive for MiTF and they were associated with an abnormal vessel. However, the commercially available antibodies for MiTF are not specific for the melanocytic MiTF isoforms [21], and a uterine leiomyoma can be positive for melanoma markers including MiTF [22]. Thus, the presence of typical features of a uterine leiomyoma ruled out PEComa. Second, vascular anomalies, particularly lymphangioma, may be involved in the differential diagnosis because of the presence of a microcystic structure. In fact, lymphangioma-like vascular change can be seen in an uterine leiomyoma [23]. The present case did not have such a vascular lesion with a D2-40-immunostained or CD31-immunostained section. In addition, an intratumoral hemorrhage was partially noted in our case; however, intratumoral congestion was not seen, and no deposits of intratumoral iron suggested that longstanding intratumoral hemorrhage was unlikely. Finally, an adenomatoid tumor was considered in the differential diagnosis. Adenomatoid tumors are usually located in the subserosa or myometrium of the uterus, and they are typically small, measuring 0.5–1 cm in diameter. Spongy cut surface may be seen in adenomatoid tumors due to dilated tumor tubules, but macroscopic cystic change of a uterine adenomatoid tumor is rare, with a reported occurrence rate of 6.7% [24]. Thus, an adenomatoid tumor clinically mimicking an ovarian tumor seemed very rare. However, there has only been one case report of a uterine adenomatoid tumor that formed a subserosal multilocular mass that radiologically mimicked a malignant ovarian tumor [25]; thus, the differential diagnosis of subserosal multilocular leiomyomas may include adenomatoid tumors and require a pathological study. Histologically, leiomyoma cells differed from adenomatoid tumor cells: the former cells have a spindle shape and eosinophilic cytoplasm, whereas the latter cells have a flat or cuboidal shape and vacuolated or pale cytoplasm. The former cells form in a fascicular fashion, whereas the latter cells typically form epithelial arrangements, including tubules and cords. Thus, it is usually easy to distinguish uterine leiomyomas from adenomatoid tumors, and an immunohistochemical study can effectively confirm the diagnosis of difficult cases. In our case, positivity for smooth muscle markers and negativity for CK5 and calretinin ruled out the presence of an adenomatoid tumor.

Thread-like bridging strands were firstly described by Hes *et al*. [26] as a histological clue of adenomatoid tumors, and they reported that all adenomatoid tumors have the morphologic feature that attenuate tumor cell cytoplasm transverse lumen. In the current case, the anastomosing, thin, cord-like arrangement of leiomyoma cells appeared to be similar to the histology of adenomatoid tumors, which may have caused a misdiagnosis of an adenomatoid tumor under low-power magnification. Such an arrangement of the leiomyoma cells was closely associated with the deposition of serous or hemorrhagic fluids, so the hydropic degeneration is possibly the unique histological feature. In fact, Coard *et al*. [27] described a case of a leiomyoma with massive hydropic degeneration similar to our case, and they reported that the hydropic degeneration resulted in multilocular and microcystic changes and marked splaying of the individual smooth muscle cells. The main pathological textbook [16] described leiomyomas with hydropic degeneration to have a delicate filigree pattern rather than thick fascicles. Thus, thread-like bridging strands, splaying, or filigree arrangement pattern of leiomyoma cells seem to indicate the same spectrum of histological architecture of a leiomyoma with hydropic degeneration. Pathologists need to be aware of the unique cellular arrangements of leiomyomas with hydropic degeneration to prevent misdiagnoses.

## Conclusions

Regardless of the unique morphology the tumor in our case, it was clinically diagnosed as an ovarian tumor. Clinical modalities, including transvaginal US, CT, and MRI failed to find the stalk of the tumor, and the results of the clinical modalities were consistent with those of an ovarian tumor. Of pathological interest, hydropic degeneration seemed to induce adenomatoid tumor-like histological change. We reviewed ovarian tumor-like leiomyomas similar to the current case, and almost all ovarian tumor-like uterine leiomyomas were subserosal, cystic, and pedunculated. Clinicians should bear in mind that a uterine leiomyoma can mimic an ovarian tumor, and it can be associated with an increased preoperative serum CA-125 level. Pathologists should also be aware that a leiomyoma with hydropic degeneration can have a histological pattern similar to that of an adenomatoid tumor. Additionally, a PEComa should not be diagnosed only by the immunoreactivity for MiTF.

## Abbreviations

CA-125: cancer antigen 125
CT: computed tomography
MiTF: microphthalmia transcription factor
MRI: magnetic resonance imaging
PEComa: perivascular epithelioid cell tumor
US: ultrasonography
WI: weighted image
